# Bibliography of Articles on Nerve Injuries, 1914-1918

**Published:** 1919

**Authors:** 


### BIBLIOGRAPHY OF ARTICLES ON NERVE INJURIES, 1914-1918.

(Editor's Note : This list makes no pretense at an exhaustive collection of titles, and it is possible in its preparation with imperfect library facilities that some of the more important papers may have been completely overlooked. It is hoped, however, that in connection with the foregoing articles it may prove to be useful to those who may be called upon to deal in the future with the many cases of peripheral nerve injury both here and at home.)

AUERBACH : Galalith zur Tubilization der Nerven nach Neurolysen und Nervennähte. *Münchener med. Wochenschr.*, 1915, n° 43.

BABINSKI, J. and FROMENT, J. : Contractures et Paralysies Traumatiques d'ordre Réflexe. *Presse Med.*, Feb., 24, 1916.

BABINSKI, J.; FROMENT, J. and HEITZ : Les Troubles Vasomoteurs et Thermiques dans les Paralysies et Contractures d'ordre Réflexe. *Annales de Médecine*, Sept.-Oct., 1916.

BELENKI : Les Symptômes Sensitifs dans les Sections Anatomiques et Physiologiques des Nerfs Périphériques. *Presse Méd.*, Feb. 17, 1916.

*BENISTY : Lésions des Nerfs : I. *Formes Cliniques des Lésions des Nerfs*, Masson et Cie, 1916. (*Trans. Univ. of London Press*, 1918.)

*BENISTY : Lesions des Nerfs : II. *Traitement et Restauration des Lésions des Nerfs* Masson et Cie, 1917. (*Trans. Univ. of London Press*, 1918.)

*BENISTY : Déformation de la Main par Blessures des Nerfs. *Nouvelle Iconographie de la Salpêtrière*, n^os^ 2 et 3, p. 65. XXVIII^e^ Année, 1916-1917.

BERNHARDT : Die Kriegsverletzungen der peripherischen Nerven. *Berliner Wochenschr.*, March, 1915.

*BOISSEAU, J.; d'ŒLSNITZ, M. : Les Mains Figées et les Pieds Bots Varus de Guerre. *Presse Médicale*, n° 15, 14 Mars, 1918.

BORCHARDT : Schussverletzungen der peripherischen Nerven. *Beitr. z. klin. Chir.*, 1915, XCVII.

BRAQUEHAYE : Section du Nerf Médian Droit. Suture Immédiate du Nerf. *Réunion médicale de la VI^e^ Armée*, Nov.-Déc. 1915.

*BUQUET, André : Plaies des Nerfs. *La Pratique de la Chirurgie de Guerre*, fasc. IV, 1916

CAMPBELL : War Injuries of the Peripheral Nerves. *Practitioner*, XCVI, 62, 1916.

CESTAN; DESCOMPS Paul, and EUZIERE, J. : Les Altérations des Empreintes Digitales dans les Lésions des Nerfs Périphériques du Membre Supérieur. *Soc. Méd. des hôpitaux.* May 5, 1916.

CESTAN and DESCOMPS, Paul : La Radiothérapie dans le Traitement de Certaine Lésions Traumatiques du Système Nerveux. *Presse Méd.*, Nov., 25, 1915.

CHAVIGNY, P. : Contractures et Mains Figées. *Paris Méd.*, II, 157-158, 1918.

CLAUDE, VIGOUROUX and DUMAS. R. Étude Anatomique de Cent Cas de Lésions Traumatiques des Nerfs des Membres. *Presse Méd.*, March 4, 1915.

*CONE, Sydney M. : Surgical Pathology of the Peripheral Nerves. *Brit. Journal of Surgery*, vol. V, n° 20, 1918.

DEBAISIEUX : Contribution à l'étude de la Technique Opératoire des Interventions sur les Nerfs Périphériques. *Travaux de l'Ambulance " Océan "*, t. 1, fasc. 1, Masson, éditeur, Paris, 1917.

*DEJERINE, M. et Mme : Quatre Schémas destinés à servir de Guide pour l'Étude des Lésions : 1° des Nerfs Périphériques ; 2° de la Moelle Épinière et de ses Racines. *Presse Médicale*, n° 17, p. 132 ; Apr. 22, 1915.

*DEJERINE : Les Indications Opératoires dans les Lésions Intrarachidiennes par Traumatisme de Guerre. *Rev. Neurologique*, July, 1915.

*DEJERINE : Considérations sur la Restauration des Nerfs Sectionnés par Projectiles de Guerre. *Bull. et Mém. de la Soc. de Chir. de Paris*, Séance du 8 Déc. 1915.

*DEJERINE, J. : Les Radiculites. *Rev. Neurologique*, Mar., 1916.

*DEJERINE, Mme : *Rev. Neurologique*, July, 1918.

*DEJERINE et MOUZON, M. J. : Contribution à l'étude des Localisations Intratronculaires des Nerfs des Membres. *Rev. Neurologique*, July, 1915.

*DEJERINE et MOUZON, M. J. : Évolution Comparée des Symptômes dans Trois Cas de Lésions du Grand Nerf Sciatique. *Rev. Neurologique*, July, 1915.

*DEJERINE et MOUZON, M. J. : Un Cas de Section du Nerf médian avec Syndrome de Restauration. *Rev. Neurologique*, Jan., 1916.

*DEJERINE, M. et Mme, et MOUZON, M. J. : Les Lésions des Gros Troncs Nerveux par Projectiles de Guerre; Les Différents Syndrômes Cliniques et les Indications Opératoires. *Presse Méd.*, May 10, July 8, Aug. 30, 1915.

*DELAGÉNIÈRE : Traitement Chirurgical des Blessures des Nerfs; Technique Opératoire et Résultats de 245 Cas de Suture et de 113 Libérations. *Bull. et Mém. Soc. de Chir. de Paris*, 1918, XLIV, 522.

DENK : Ueber Schussverletzung der Nerven. *Beitr. z. klin. Chir.*, 1914, XCI.

DOFFNER : Zur Methodik der Naht der peripheren Nerven. *München. med. Wochenschr.*, 1915, LXII.

*DEPAGE : Les Interventions sur les Nerfs Périphériques et leurs Suites Éloignées. *Comptes Rendus de la Conférence chir. Interalliée*, 3^e^ Session, 5-8 Nov. 1917.

DUMAS : *Bull. et Mém. de la Soc. Chir. de Paris*, t. XLIII, p. 1184.

*Dumas, René : Libération des Nerfs et Recupération Fonctionnelle. *Bulletins et Mém de la Soc. de Chir. de Paris*, Feb. 8, 1916.

Duroux, E. and Couvreur, E. : Experimental and Clinical End Results of Nerve Suture. *Rev. de Chir.*, LIII, 401, Paris, 1917.

Dustin : Le Service de Neurologie à l'Ambulance de l'Océan. *Travaux de l'Ambulance "Océan"*, t. I, fasc. 1, Masson, éditeur, Paris, 1917.

*Dustin : Les Lésions Post-traumatiques des Nerfs; Contribution à l'Histopathologie du Système Nerveux Périphérique chez l'homme. *Travaux de l'Ambulance "Océan"*, t. I, fasc. 1, Jul., 1917.

*Dustin : Les Scléroses Ischémiques Localisées du Membre Inférieur. *Trav. de l'Amb. "Océan"*, t. I, fasc. 1, Jul., 1917.

Duval : *Bull. et Mém. de la Soc. Chir. de Paris*, 1915.

Ferranini : Contributo allo Studio delle Lesioni del Simpatico nelle Ferite dei Nervi degli Arti. *Riforma Med.*, 33, 226-232, and 253-256, 1917.

Foix : *Rev. Neur.*, p. 904, June, 1916.

Froment : La Préhension dans les Paralysies du Nerf Cubital. *Presse Méd.*, nº 50, p. 409, Oct. 21, 1915.

Fullerton : The Use of a Sleeve of Veins in Nerve Suture. *Brit. Med. Jour.*, Aug., 1915.

Gerulanos : Schussverletzungen der peripheren Nerven aus dem Balkankrieg *Beitr. z. klin. Chir.*, 1914, XCI.

*Gosset : Sur les Blessures des Nerfs. *Comptes Rendus de la Conférence Chirurgicale Interalliée.*, 4e session, 11-15 Mars, 1918.

Gratz : Schussverletzungen peripherer Nerven. *Beitr. z. klin. Chir.*, 1915, XCVII.

Grosse : Schussverletzungen peripherer Nerven. *Beitr. z. klin. Chir.*, 1915, XCVII.

Guillain, Georges, and Barré, A. : Les Contractures dans la Pathologie Nerveuse de Guerre. *Bulletin de la Soc. Méd. des Hôpitaux*, Jan., 21, 1916.

Hans : Naht durchtrennter Nerven mittels Einhulsen in Eigengewebe. *Zentralblatt f. Chir.*, 1915, nº 45.

Heekes, J. W. : Case of Prompt Recovery after Nerve Suture. *B. M. J.*, 1, 507, 1918.

Heile and Hezel : Unsere bisherigen Erfahrungen bei der Behandlung im Kriege verletzter peripherer Nerven. *Beitr. z. klin. Chir.*, 1915, XCVI.

Hirschel : Erfahrungen über Schussverletzungen der Nerven. *Deutsche Zeitschrift f. Chir.*, 1914-1915, CXXXII.

Hofmeister : Ueber doppelte und mehrfache Nervenpfropfung. *Beitr. z. klin. Chir.*, 1915, XCVI.

Hughes, D. M. : Anomalous Muscular Action in Nerve Injuries. *Brit. Med. Jour.* 1, 720, 1918.

Ingerbigsten : A Contribution to the Biology of Peripheral Nerves in Transplantation. *Jour. Exper. Med.*, 1915, XXII.

*Jones, Rob. : Abstracts from Alder Hey Orthopedic Hosp.; Lesions of Peripheral Nerves Resulting from War Injuries; Pathology and Treatment. *Brit. Med. Jour.*, March 30, 1918.

*Jones : Suture of Nerves, and Alternative Methods of Treatment by Transplantation of Tendon. *Notes on Military Orthopedics*, Chap. II, Cassell and Co., Ltd., 1917.

Jourdon and Sicard : Étude Macroscopique et Microscopique des Lésions des Nerfs par Blessure de Guerre. *Presse Méd.*, July 29, 1915.

Kirk and Dean Lewis : Regeneration in Peripheral Nerves. *Bulletin Johns Hopkins Hosp.*, 1917, XXVIII.

Kredel : Ueber das Verhalten der auf Operierte Schussverletzte Nerven Ueberpflanzten Fascienlappen. *Zentralblatt f. Chir.*, 1912, nº 13.

*Laignel-Lavastine et Courbon, Paul : Seize Déformations Paratoniques de la Main Consécutives aux Plaies de Guerre. *Nouvelle Iconographie de la Salpêtrière*, XXVIIIe Année, nos 2 and 3, 1916-1917.

*Langley, J. N. : On the Separate Suture of Nerves in Nerve Trunks. *Brit. Med. Jour.*, 1.º 2976, p. 45, Jan. 12, 1918.

Langley : The Effect of Intermittent Stretching on Muscles and Nerves after Nerve Severance. *Ibid.*, Feb. 2, 1918.

LANGLEY and HASHIMOTO : On the Suture of Separate Nerve Bundles in a Nerve Trunk and on Internal Nerve Plexuses. *Jour. Physiol.*, p. 318, Sept., 12, 1917.
*LERI, André : Quelques Déformations des Mains et des Pieds chez les " Blessés Nerveux ". *Ibid.*
LERICHE : *Presse Méd.*, p. 178, 1916.
*LERICHE : De la Sympathectomie Péri-artérielle et de ses Résultats. *Presse Méd.*, nº 50, Sept., 1917.
LORTAT-JACOB, and HALLEZ, G. L. : Treatment of Causalgia of the Median by Ligating the Nerve with Catgut. *Bull. et Mém. Soc. Méd. des Hôp. de Paris*, XLII-239, 1918.
MARIE, P. and FOIX : Indications Opératoires fournies par l'Examen Histologique des Nerfs Lésés par Plaies de Guerre. *Presse Méd.*, Jan., 30, 1916.
MARIE, P., and BENISTY, Mme : Du Retour de la Contractilité Faradique avant le Rétablissement de la Motilité Volontaire dans les Muscles Paralysés, à la Suite des Lésions des Nerfs Périphériques. *Soc. de Neur. de Paris*, Apr., 15, 1915, and May 6, 1915; *Rev. Neur.*, May-June, 1915, p. 494, p. 557, July, 1915.
MARIE, P., and FOIX : *Presse Méd.*, nº 6, Jan. 1916 (cf. above).
MARIE, P. and MEIGE, H. and GOSSET, A. : Les Localisations Motrices dans les Nerfs Périphériques. *Acad. de Médecine*, Déc., 28, 1915.
MARIE, P. and FOIX : Sur une Forme Spéciale de " Parésie Paratonique des Muscles Moteurs de la Main ". *Soc. Méd. des Hôpitaux*, Feb., 4, 1916.
MARIE, P. and BENISTY, Mme : Individualité Clinique des Nerfs Périphériques. *Soc. de Neur.*, Mar., 15, 1915; *Rev. Neur.*, May-June, 1915.
MARIE, P. and BENISTY, Mme : Une Forme Douloureuse des Lésions du Nerf Médian par Plaie de Guerre. *Acad. de Méd.*, Mar., 16, 1915.
MARINESCO, G. : The Characteristics of Amputation Neuromata. *Proc. Roy. Soc. Med.*, XI, 5, 1918.
*MEIGE, M. Henry, and others : Sutures Nerveuses. Séance de la Soc. Neur. du 20 Mars 1918. *Revue Neurologique*, XXVᵉ Année, p. 262, Mar.-April, 1918.
MEIGE, H. : *Soc. de Neur. de Paris*, Apr., 7, 1916. Cf. *Rev. Neur.*, Apr.-May, 1916 (p. 499 and following).
MEIGE, H. and BENISTY, Mme : Les Formes Douloureuses des Blessures des Nerfs. *Soc. de Neurol.*, July, 1, 1915.
MEURIOT, H. and PLATON : One Hundred Cases of Liberated Nerves in Rubber Sheaths. *Bull. et Mém. Soc. de Chir. de Paris*, XLIV, 850, 1918.
MOUCHET : *Bull. et Mém. de la Soc. de Chir. de Paris*, 1915.
*MOYNIHAN, Berkeley : An Address on Injuries to the Peripheral Nerves and their Treatment. *Brit. Med. Jour.*, Nov. 3, 1917.
NAGEOTTE : Nerve Grafts. *Soc. de Biol.*, May, 1917.
NAGEOTTE : Nerves and Scar. *Ibid.*, June, 1917.
NAGEOTTE : *Comptes Rendus de la Soc. de Biol.*, vol. LXXXIII, and *Rev. Neur.*, Jul., 1915.
NAGEOTTE : Le Processus de la Cicatrisation des Nerfs. *Rev. Neur.*, July, 1915.
NONNE : Ueber Kriegsverletzungen der peripheren Nerven. *Med. Klin.*, 1915.
ŒCONOMAKIS : Ueber Traumatische Lähmungen der peripheren Nerven nach Schussverletzungen. *Neurol. Centralbl.*, 1914, XXXIII (Balkan War).
PITRÈS : *Rev. Neurologique*, p. 489, Apr.-May, 1916.
PITRES : Sur les processus histologiques qui président à la cicatrisation et à la restauration fonctionnelle des nerfs traumatisés. *Jour. de Médecine de Bordeaux*, Déc., 1915.
PITRES : La Valeur des Signes Cliniques Permettant de Reconnaître dans les Blessures des Nerfs Périphériques : *a*) la Section Complète du Nerf; *b*) la Restauration Fonctionnelle. *Soc. de Neur.*, April, 6, 1916; *Rev. Neur.*, p. 477, Apr.-May, 1916.
REICHMANN : Die Schussverletzung Peripherischer Nerven. *Deutsch. Med. Wochenschr.*, 1915.
ROJAS, P. : Experimental Degeneration and Regeneration of Peripheral Nerves. *An. d. Inst. d. Clin. Med.*, Buenos-Aires, II, 218, 1917.

*Roussy, G., Boisseau, J., d'Œlsnitz, M. : Les Acro-contractures et les Acro-paralysies. Mains Figées et Pieds Bots Varus Psychonévrosiques, leur Nature, leur Traitement.

Sicard : Traitement de la Névrite Douloureuse du Médian par l'Alcoolisation Tronculaire Sus-lésionnelle. *Soc. Méd. des Hôp.*, July, 9, 1915.

Sicard : Traitement de Certaines Algies et Acro-contractures Rebelles par l'Alcoolisation Nerveuse Locale. *Ibid.*, Déc., 17, 1915.

Spielmeyer : Zur Frage der Nervennaht. *Münchener Med. Wochenschr.*, 1915, LXII.

Spriggs, N. I.; Clarke, A. V. : Ulnar Nerve Paralysis; Operative Treatment for the Disability of Main-en-griffe. *Lancet*, London, CXCIV, 802, 1918.

Steinthal : Die Deckung grosserer Nervendefekte durch Tubularnaht. *Beitr. z. klin. Chir.*, 1915, XCVI.

Stierlin and Vischer : *Deutsche Zeitschr. f. Chir.*, 1914, CXXXI (Balkan War).

Stoffel : Ueber Nervenmechanik und ihre Bedeutung für die Behandlung der Nervenverletzungen. *Münchener Med. Wochenschr.*, 1915, LXII.

Stoffel : Ueber die Behandlung verletzter Nerven im Krieg. *Münchener Med. Wochenschr.*, 1915, LXII.

*Stookey, Byron : Surgical Considerations of Peripheral Nerve Injuries. *Surg., Gynec.* and *Obstet.*, vol. XXVII, p. 362, Oct., 1918.

Tanton : *Bull. et Mém. de la Soc. de Chir. de Paris*, p. 1633, July, 24, 1917.

Thole : Kriegsverletzungen Peripherer Nerven. *Beitr. z. klin. Chir.*, XCVIII, 1915. (Good review of recent literature).

Thomas, André : La Sensibilité Douloureuse de la Peau à la Piqûre et au Pincement dans la Période de Restauration des Nerfs Sectionnés, après Suture ou Greffe. *Soc. de Neur.*, Feb., 3, 1916; *Rev. Neur.*, p. 311, Feb., 1916.

Tinel : Les Blessures des Nerfs. *Sémiologie des Lésions Nerveuses Périphériques par Blessures de Guerre*, Masson et Cie, 1916.

Tinel : Le Signe du " Fourmillement " dans les Lésions des Nerfs. *Presse Méd.*, Oct., 7, 1915.

Tinel : Causalgia of Median Nerve; Insufficiency of Peri-arterial Sympathectomy. *Rev. Neurol.*, Paris, XXV, 79, 1918.

Tuffier : *Bull. et Mém. de la Soc. de Chir. de Paris*, 1916.

Villaret, Maurice : Contribution à l'étude des Troubles du Système Pileux et de la Sudation Spontanée des Membres au Cours des Lésions Traumatiques de Guerre des Nerfs Périphériques. *Soc. Médicale des Hôpitaux*, Déc., 17, 1915; and *Rev. Neur.*,. p. 495, Apr.-May, 1916.

White, J. R. : Operative Treatment of Injuries of the Peripheral Nerves. *Brit. Med. Jour.*, 388, Mar., 1917.

Zimmern, A. : Quels Enseignements Nous Fournit la Réaction de Dégénérescence dans les Blessures des Nerfs. *Presse Med.*, Apr., 15, 1915.

